# eRNAs Identify Immune Microenvironment Patterns and Provide a Novel Prognostic Tool in Acute Myeloid Leukemia

**DOI:** 10.3389/fmolb.2022.877117

**Published:** 2022-05-02

**Authors:** Ziming Jiang, Junyu Long, Kaige Deng, Yongchang Zheng, Miao Chen

**Affiliations:** ^1^ Department of Hematology, Peking Union Medical College Hospital, Peking Union Medical College and Chinese Academy of Medical Sciences, Beijing, China; ^2^ Eight-Year MD Program, Peking Union Medical College and Chinese Academy of Medical Sciences, Beijing, China; ^3^ Department of Liver Surgery, Peking Union Medical College Hospital, Chinese Academy of Medical Sciences and Peking Union Medical College, Beijing, China

**Keywords:** acute myeloid leukemia, enhancer RNA, non-coding RNA, bone marrow microenvironment, immunotherapy, ICI therapy, prognosis

## Abstract

**Background:** Enhancer RNAs (eRNAs) play an essential role in tumorigenesis as non-coding RNAs transcribed from enhancer regions. However, the landscape of eRNAs in acute myeloid leukemia (AML) and the potential roles of eRNAs in the tumor microenvironment (TME) remain unclear.

**Method:** Gene expression data collected from The Cancer Genome Atlas (TCGA) project were combined with Histone ChIP-seq so as to reveal the comprehensive landscape of eRNAs. Single-sample gene set enrichment analysis algorithm (ssGSEA) and ESTIMATE were employed to enumerate immune cell infiltration and tumor purity.

**Results:** Most prognostic eRNAs were enriched in immune-related pathways. Two distinct immune microenvironment patterns, the immune-active subtype and the immune-resistant subtype, were identified in AML. We further developed an eRNA-derived score (E-score) that could quantify immune microenvironment patterns and predict the response to immune checkpoint inhibitor (ICI) treatment. Finally, we established a prognostic nomogram combining E-score and other clinical features, which showed great discriminative power in both the training set [Harrell’s concordance index (C index): 0.714 (0.651–0.777), *p* < 0.0001] and validation set [C index: 0.684 (0.614–0.755), *p* < 0.0001]. Calibration of the nomogram was also validated independently.

**Conclusion:** In this study, we systematically understood the roles of eRNAs in regulating TME diversity and complexity. Moreover, our E-score model provided the first predictive model for ICI treatment in AML.

## Introduction

Acute myeloid leukemia (AML) is the most common leukemia of the hematopoietic system (De Kouchkovsky and Abdul-Hay, 2016; Estey, 2018), which is characterized by rapid proliferation and invasion of immature myeloid cells in the bone marrow (BM) and peripheral blood (PB), resulting in hematopoietic failure of normal blood cells. Chemotherapy and hematopoietic stem cell transplantation are the first-line treatment for AML patients (Döhner et al., 2010; De Kouchkovsky and Abdul-Hay, 2016; Tyner et al., 2018a; Estey, 2018), though only one-third of adult AML patients could get durable remission (Vago and Gojo, 2020). A high risk of relapse is considerable even after remission (Estey, 2018; Thol and Ganser, 2020). However, the cellular and molecular characteristics of AML have been investigated to promote current therapies (Yang and Wang, 2018; Vago and Gojo, 2020). For example, high counts of regulatory T cells were observed in both PB and BM of AML patients and inhibited effector T cells more effectively than in control (Szczepanski et al., 2009; Williams et al., 2019). Significantly, the immunosuppressive microenvironment of BM was closely associated with a poor prognosis (Vago and Gojo, 2020), which provided new insights into therapeutic targets. The combination of ICI treatment and chemotherapy showed great advantages. A more prolonged median overall survival (OS) of 6.3 months was yielded in Nivolumab combined with azacitidine group, compared with the azacitidine group in 70 older, relapsed or refractory (R/R) AML patients (Daver et al., 2019). Another PD1-blocking antibody, pembrolizumab, with decitabine or azacitidine, showed a similar response and survival advantage in R/R AML patients (Lindblad et al., 2018; Gojo et al., 2019). Nonetheless, a randomized phase II study of durvalumab with azacytidine verse azacytidine alone did not show a significant advantage in 214 newly diagnosed AML patients (Zeidan et al., 2019), suggesting new models to predict the response of immunotherapy are underexplored.

eRNAs are transcribed in specific enhancer regions and considered as non-coding RNAs. It has been widely proven that eRNAs play essential roles in tumor proliferation, invasion, and migration. Several eRNAs, such as NET1e in breast cancer (Zhang et al., 2019) and CCAT1 in squamous cell carcinoma cells (Jiang et al., 2018), showed strong interactions with target genes and contributed to cancer progression. Recent studies identified eRNAs as a better marker for active enhancers than H3K27ac, the histone modification mediated by P300 (Tyssowski et al., 2018). Due to their highly tissue-specific and disease state-specific characteristics, eRNAs have been demonstrated as an excellent marker to identify phenotypes such as ICI treatment response compared with mRNAs (Chen and Liang, 2020), which has been validated in a broad range of diseases including lung cancer (Qin et al., 2020; Ma et al., 2021), glioblastoma (Guo et al., 2021), and hepatocellular carcinoma (Cai et al., 2021). However, systematic studies for eRNAs in AML are still under exploration.

Zhang et al. (2019) established a systematic strategy for investigating eRNA in the RNA-seq of large patient cohorts. The FANTOM5 database has generated approximately 65,000 high-quality and *in vivo*-transcribed enhancer loci with CAGE-seq in hundreds of primary cells and cell lines (Andersson et al., 2014). The ENCODE and Blueprint databases provided a wealth of histone modification locus information, including H3K4me1, H3K4me3, and H3K27ac annotation (Inoue et al., 2017). The TCGA, Beat AML, and GEO databases provided detailed data on gene expression profiles and clinical annotations of AML patients (Weinstein et al., 2013; Tyner et al., 2018b). In conclusion, comprehensive high throughput sequencing provided detectable methods to identify eRNAs in AML. We screened the TCGA database to identify eRNAs closely correlated with clinical prognosis and TME abundance, thereby exploring the characteristics of different immune subtypes associated with eRNAs in AML patients. A scoring system was established via machine learning to investigate the association between immune microenvironment patterns and existing clinicopathological stratifications, demonstrating that the TME can supplement the current risk classification. As we expected, the scoring system named E-score was found to correlate with the response to anti-PD1/PDL1 agents computationally. Finally, we developed a clinical prognostic model for AML patients based on the E-score, which showed accurate predictive power.

## Methods

### Data Collection and Preprocessing

We downloaded RNA-seq BAM files, mRNA expression matrixes, and clinical features of acute myeloid leukemia (TCGA-LAML) from the TCGA data portal (https://portal.gdc.cancer.gov/) (Weinstein et al., 2013). The validation datasets were downloaded from the Beat AML (http://www.vizome.org/) (Tyner et al., 2018a) and Gene Expression Omnibus (GEO) database (https://www.ncbi.nlm.nih.gov/geo/, GSE37642 (Li et al., 2013), GSE12417 (Metzeler et al., 2008), and GSE10358 (Tomasson et al., 2008)). To ensure the stability of the prediction, patients with survival time <1 month were excluded. The PRO-seq BAM files, Histone ChIP-seq, and P300 ChIP-seq data of the Kasumi-1 cell line were downloaded from the GEO database (GSE83660 and GSE100446) (Zhao et al., 2016; Xu et al., 2019). From the Encyclopedia of DNA Elements (ENCODE) portal (https://www.encodeproject.org/), we downloaded the Hi-C interactions data of K562 and GM12878 cell lines with the following identifiers: ENCFF996XEO and ENCFF355OWW (Inoue et al., 2017). The Histone ChIP-seq data of the mononuclear cells of the bone marrow were collected from the Blueprint project (IHEC portal: https://epigenomesportal.ca/ihec/index.html, IHECRE00000277) (Adams et al., 2012; Stunnenberg et al., 2016). The ribosome-deleted RNA-seq data of two AML patients’ BM samples were obtained from GSE87285 (Loke et al., 2017). All detailed information on datasets mentioned above is listed in supplementary table 1.

### Quantification and Annotation of eRNA

Annotation of enhancer loci was downloaded from the FANTOM5 project (http://fantom.gsc.riken.jp/5/) (Andersson et al., 2014). We filtered out eRNA loci overlapping with known annotated elements and 1 kb extension from both the transcription start site (TSS) and transcription end site (TES) to eliminate introns, exons, promoters, rRNA repeats, and other transcribed elements. The 3 kb of the middle locus of the enhancer was designated as the potential eRNA region (Zhang et al., 2019). RNA-seq data of the TCGA-LAML cohort were mapped to eRNA regions by featureCounts and normalized to reads per million (RPM) (Liao et al., 2014). eRNA with RPM ≥1 in at least one sample was defined as an active eRNA (Zhang et al., 2019; Zhang et al., 2021). The enhancer loci from all the human tissues and cell lines in the database were included in the analysis. Liftover was used to convert genomic regions from various genomic versions to the hg19 genome version (Rhead et al., 2010). The annotation of enhancer loci was supported by R package annotatr (Cavalcante and Sartor, 2017). The histograms of promoters, enhancers, and random loci were created by HOMER (http://homer.ucsd.edu/homer/) (Heinz et al., 2010). Immune-related eRNAs (ir-eRNAs) were defined as at least correlated with an immune cell type (absolute Spearman’s correlation Rs > 0.3, *p* < 0.05). We used univariate Cox regressions (*p* < 0.01, hazard ratio (HR) > 1.1 or <0.9) to identify prognostic eRNA.

### Identification of Enhancer–Gene Pair and Validation of Chromatin State Analysis

The pair of eRNAs and protein-coding genes was identified based on close distance (1 MB) and the corresponding expression level (absolute Spearman’s correlation Rs > 0.3 and FDR <0.05) concurrently (Zhang et al., 2019). We selected the defined eRNA loci, promoter of protein-coding genes, and random loci of the genome (0.5–4.5 kb away from the region of the defined enhancer and excluded any overlapping annotation) to exhibit the genome coverage. The Histone and P300 ChIP signal was visualized by Deeptools, and the HiC data were illustrated by HiCExplorer (Wolff et al., 2020).

### Unsupervised Clustering Analysis

To investigate distinct patterns, we used the R package ConsensusClusterPlus for unsupervised clustering analysis (Wilkerson and Hayes, 2010). The number of iterations was set at 1,000 to ensure clustering stability. The other parameters, clusterAlg, distance, and linkage, were set as pam, Pearson, and ward.D.

### Gene Set Variation Analysis, Gene Set Enrichment Analysis, and Gene Ontology Annotation

GSVA is a nonparametric, unsupervised method for assessing alternations in the activity of signaling pathways and biological processes using expression datasets. We compared signaling pathways across different ir-eRNA patterns using the R package GSVA (Hänzelmann et al., 2013). The gene set “c2. cp.kegg.v6.2. symbols” from the Molecular Signatures Database (MSigDB) was used as the predetermined dataset. Statistical significance was defined as an adjusted *p* value of less than 0.05. We used the R package clusterProfiler to annotate the ir-eRNA-related gene for Gene Ontology (GO) analysis by biological processes (BPs) (*p* < 0.05, FDR <0.05) and the differentially expressed genes (DEGs) for gene set enrichment analysis (GSEA) by the Kyoto Encyclopedia of Genes and Genomes (KEGG) with a default parameter.

### Evaluating Cell Infiltration in the TME

By generating a gene set using cell-specific markers, single-sample gene set enrichment analysis (ssGSEA) allows us to assess the number of immune cells in AML BM samples. A previous study yielded gene sets for labeling each kind of TME-infiltrated immune cells, such as activated dendritic cells, activated CD8^+^ T cells, macrophages, active CD8 T cells, and regulatory T cells (Barbie et al., 2009; Charoentong et al., 2017). The relative abundance of TME cells in each sample was represented by the estimated abundance score *in silico*. In addition, the ESTIMATE method was also used to compute immune and stromal scores, as well as tumor purity, corroborating the ssGSEA approach.

### Identifying DEGs in Different ir-eRNA Patterns

Based on prognostic ir-eRNAs, AML patients were split into two different expression patterns. To determine pattern-related genes, we used the R package limma to compare the expression profiles between the two patterns. An empirical Bayesian algorithm using moderated t-tests was used to carry out this strategy. The adjusted *p* value of 0.05, determined with Benjamini–Hochberg correction, was set as the significant criteria for detecting DEGs (Ritchie et al., 2015).

### Generating Gene Signature and E-Score

We developed a scoring system to assess the immune pattern of individual AML patients. First, we standardized the expression of DEGs recognized from different immune subtypes in all TCGA samples. Patients were divided into two subgroups by DEGs through unsupervised clustering analysis. Next, we used a univariate Cox regression model for prognostic analysis of each gene in the signature (*p* < 0.01, HR > 1.1 or <0.9). We calculated the correlation between different subtypes and signature genes (Pearson correlation analysis). The positively correlated genes were defined as gene set A, and the negatively correlated genes were defined as gene set B. We then performed principal component analysis (PCA) to construct pattern-related signatures. The benefit of this strategy was that it concentrated the scores on the dominant network of gene signatures while downweighting contributions from genes that did not correlate with other set members. Principal component 1 of each gene set was extracted as PC1. A and PC1.B to calculate E-score, which was defined in an approach similar to that of the Genomic Grade Index (GGI) (Van 'T Veer et al., 2002; Zeng et al., 2019):

$$E\_score=\sum \left(PC1.A_{i}+PC1.B_{i}\right)\;,$$

where i is the expression of a gene related to each signature gene set.

### Statistical Analysis

Spearman’s rank correlation analysis evaluated the correlation coefficient between the expression of ir-eRNAs and TME-infiltrated immune cells (Sokol et al., 2017). We utilized the R package survminer to identify the cutoff point between each E-score subgroup based on the connection between the E-score and the overall survival of the patients. To get the maximum rank statistic, all possible cutoff points were evaluated repeatedly. Patients were separated into high- and low-E-score groups using log-rank statistics to minimize batch effects for maximum selection (Kerschke et al., 2020). The Kaplan–Meier curves were used to create survival curves for prognostic analysis, and the significance of differences was determined using the log-rank test. The hazard ratios (HRs) and *p* values of ir-eRNAs and signature genes were calculated using a univariate Cox regression model. To find independent prognostic factors, we employed a multivariate Cox regression model. The R package forestplot was applied to visualize the results of univariate and multivariate Cox regression. Receiver operating characteristic (ROC) curves were used to assess the sensitivity and specificity of E-score, and the area under the curve (AUC) was determined using the R package survivalROC. The R package RCircos was used to illustrate the chromosomal location of the ir-eRNAs and target genes. The significance criterion for all statistical comparisons was 0.05, and all comparisons were two sided. R 4.0.2 was used to conduct all statistical analyses.

### Availability of Data and Source Code

All expression profiles and clinical annotation files were downloaded from public databases. Our code is available on request.

## Results

### The Landscape of eRNAs in Acute Myeloid Leukemia

**FIGURE 1:**
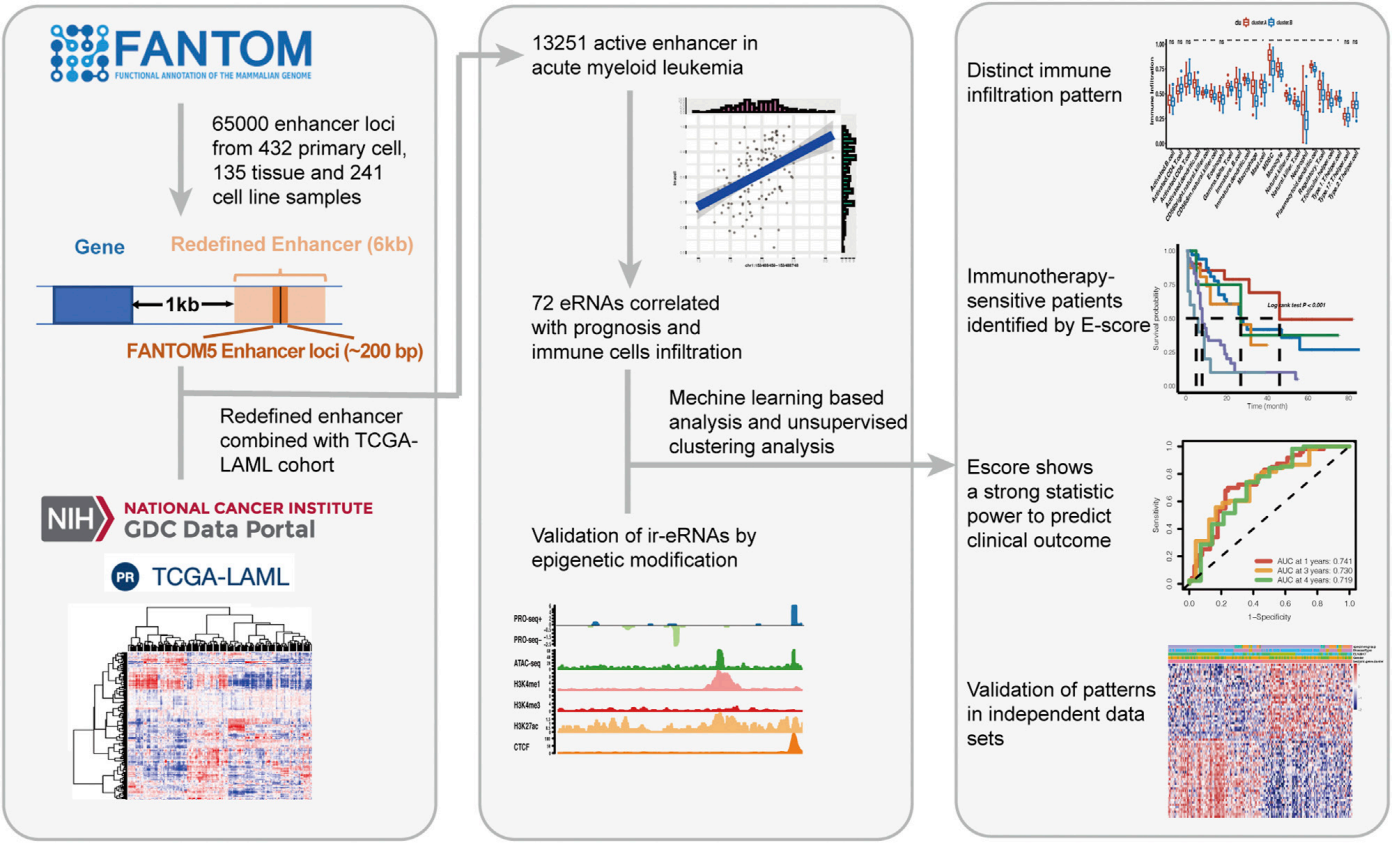
Overview of the study.

**FIGURE 2:**
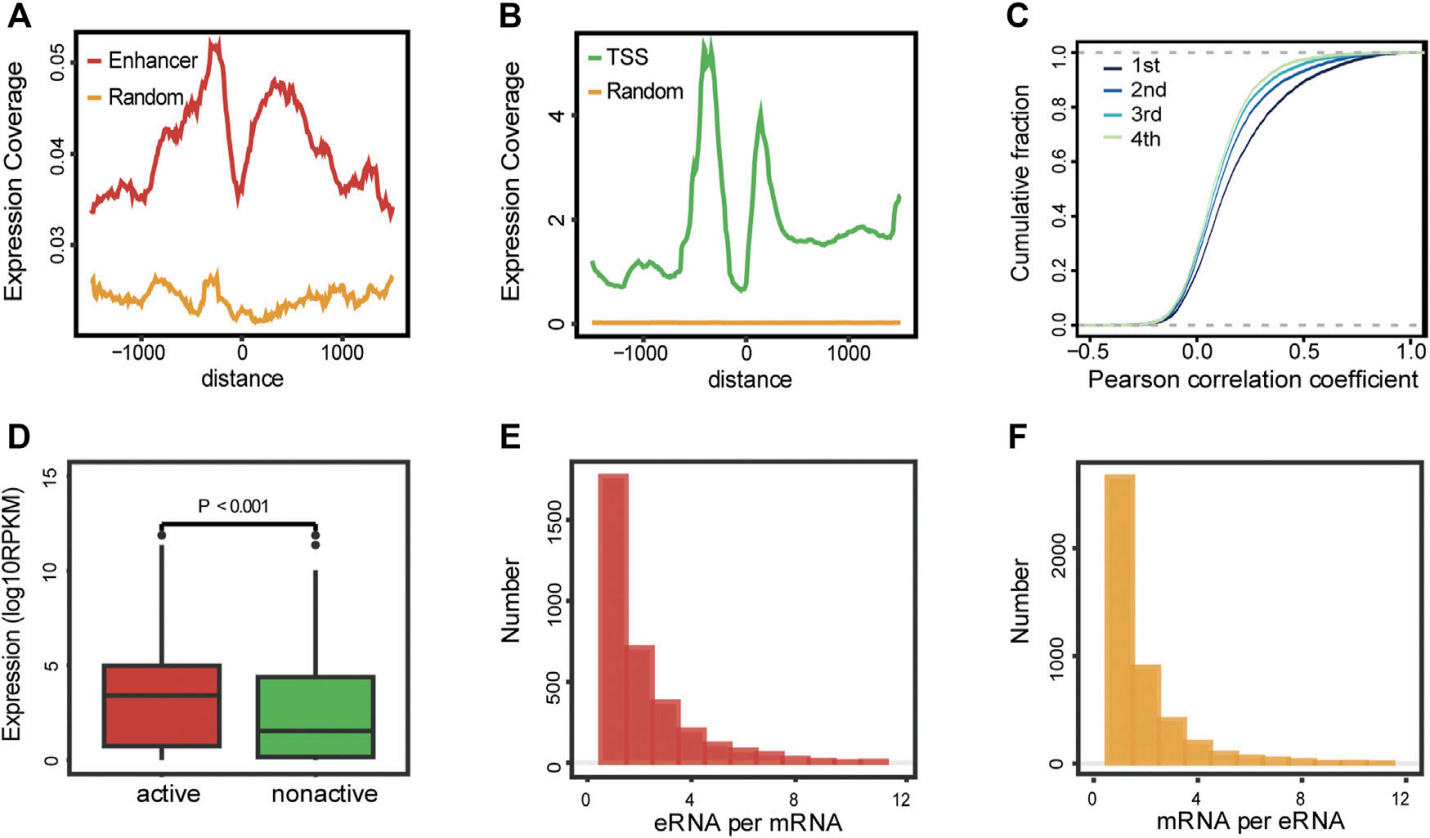
Landscape of eRNAs in AML. **(A–B)** Expression coverage of enhancers **(A)** and protein-coding genes **(B)** in AML patients. TSS: transcription start site of the protein-coding gene; enhancer: enhancer loci from FANTOM5 project; and random: random loci. **(C)** Pairwise Pearson correlation analysis of detectable eRNAs and their nearby genes. 1st, the closest gene, 2nd, the second closest gene, 3rd, the third closest gene, and 4th, the fourth closest gene. **(D)** Expression levels of protein-coding genes near active and nonactive eRNAs (Wilcoxon test, *p* < 0.001). **(E)** Distribution of the number of protein-coding genes co-expressed with per eRNA. **(F)** Distribution of the number of eRNAs co-expressed with per protein-coding genes.

The overview of the study is depicted in Figure 1. We started by gathering approximately 65,000 enhancer loci from the FANTOM5 project, a robust enhancer database including enhancers from most human tissues and cell lines (Andersson et al., 2014). Then, we identified 13,251 enhancer RNAs in the TCGA-LAML cohort, none of which overlapped with annotated genomic regions. Active enhancer loci were typically described as eRNA transcription and unique epigenomic modification patterns, such as high H3K4me1, high H3K27ac, low H3K4me3, and high P300 levels (Klemm et al., 2019). To verify the reliability of enhancer loci in AML, we then investigated the epigenomic modification patterns of 13,521 eRNA regions, which showed enhancer-like epigenomic patterns compared with random loci and protein-coding gene loci (Supplementary Figure S1). In addition, the distribution of RNA transcription abundance revealed obvious transcription peaks in a classic manner among enhancer regions, demonstrating that the expression of enhancer regions was not the transcription background noise (enhancer loci vs. random loci, Figure 2A). On average, the expression level of eRNAs was less than that of protein-coding genes (enhancer loci vs. random loci and transcription start site (TSS) of protein-coding gene vs. random loci, Figures 2A,B). Enhancers usually play roles as cis-elements (Klemm et al., 2019). Previous studies revealed that eRNAs were excellent markers for active enhancers, so eRNAs usually correlated with adjacent genes. To illustrate the characteristics of eRNAs, pairwise Pearson correlation analysis revealed that eRNAs were more correlated with neighboring genes in a distance-related way, which indicated that eRNA has a stronger association with the closest gene than distal ones (Figure 2C). The closest coding genes near active eRNAs (RPM >1) exhibited a higher expression level than those near nonactive eRNAs (*p* < 0.001, Wilcoxon test; Figure 2D). Similar to previous studies, we observed that most eRNAs were mapped to interact with one adjacent gene (51.20%, 1,171/3,459, Figure 2E), and most genes were only matched to a single eRNA as well (59.74%, 2,664/4,459, Figure 2F). In conclusion, we demonstrated the reliability of the defined enhancer loci and presented the landscape of eRNA in AML. Our results and recent studies suggested that eRNA levels were intimately associated with the expression of nearby genes (Andersson et al., 2014; Chen and Liang, 2020).

### Prognostic ir-eRNAs Correlated With the Tumor Microenvironment in the Bone Marrow

**FIGURE 3:**
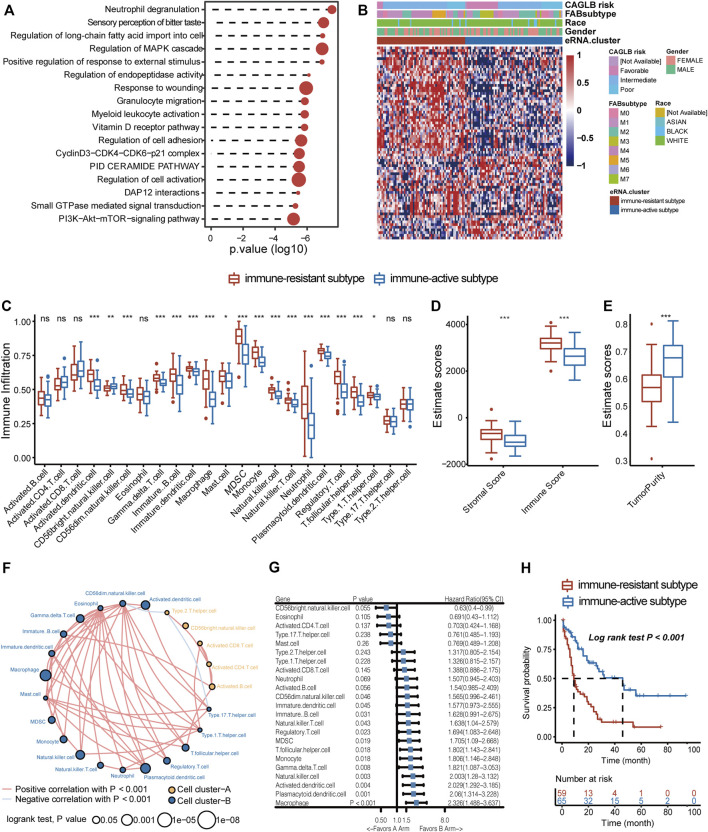
ir-eRNAs regulate the development of the tumor microenvironment in the bone marrow. **(A)** GO analysis of 258 prognostic eRNAs showed immune-related functional activation. **(B)** Two eRNA clusters showed different clinical and gene expression characteristics, illustrated by a heatmap. **(C)** Analysis of the immune infiltration level of 23 immune cell subtypes in two eRNA clusters (ns, *p* > 0.05, *p < 0.05; **p < 0.01; ***p < 0.001, Wilcoxon test). **(D)** ESTIMATE score of stromal, immune components in the TME in two eRNA clusters (Wilcoxon test, ***p < 0.001). **(E)** ESTIMATE score of tumor purity in two eRNA clusters (Wilcoxon test, ***p < 0.001). **(F)** Cell clustering revealed correlations among 23 immune cell subtypes (Spearman correlation, *p* < 0.001, log-rank test, *p* < 0.05). **(G)** Univariate Cox regression showed prognostic relevance of different immune cell infiltrations. **(H)** Survival analysis showed different survival outcomes between two eRNA clusters (log-rank test *p* < 0.001).

We then concentrated on eRNAs correlated with prognosis, which may play crucial roles in tumor progression and proliferation. We filtered out 258/13,251 prognostic eRNAs (Supplementary Table S2) by univariate Cox regression. To explore the potential functions of prognostic eRNAs, co-expression analyses were performed. Correlated mRNAs in the 1 Mb region were identified as potential target genes (Rs > 0.3, Spearman correlation analysis). Functional enrichment analysis revealed that the bitter taste-related pathway and several vital oncogenic signaling pathways such as PI3K-Akt-mTOR and MAPK were enriched in GO enrichment analysis (Figure 3A) (Nepstad et al., 2020; Kim et al., 1999). Surprisingly, prognostic eRNAs were most concentrated on immune-related pathways, such as myeloid leukocyte activation and granulocyte migration (Figure 3A). Therefore, we suggested that AML blasts shape immune microenvironment patterns through eRNAs and related genes. We evaluated the immune cell abundance of each sample by ssGSEA and filtered 72 eRNAs correlated with immune cell abundance (Supplementary Figure S2), which were defined as ir-eRNAs. Due to the diverse expression of ir-eRNAs in different samples, we explored whether ir-eRNAs exhibited distinct expression patterns in AML. Unsupervised clustering analysis was performed, and two clusters were identified (Supplementary Figures S3A–E, Figure 3B). One cluster defined as an immune-resistant subtype showed a higher infiltration level of suppressive cells, including dendritic cells, macrophages, regulatory T cells, and myeloid-derived suppressive cells (MDSCs). The other cluster defined as the immune-active subtype showed a lower level of suppressive cells and a trend of a higher level of activated T cells without statistically significant at the 5% level (Figure 3C). The stromal and immune scores determined by ESTIMATE were also higher in the immune-resistant subtype (stromal score, *p* < 0.001, Wilcoxon test; immune score, *p* < 0.001, Wilcoxon test; Figure 3D), while the immune-active subtype had a higher tumor purity (*p* < 0.001, Wilcoxon test; Figure 3E), in line with the work of Yan et al. (2019). Cell clustering revealed complicated correlation networks of 23 immune cells and their prognostic significance by the log-rank test (Figure 3F). In addition, Figure 3G shows the detailed hazard ratio of each immune cell by univariate Cox regression analysis. Plasmacytoid dendritic cell and macrophage, previously reported as cancer-promoting cells, had hazard ratio >1 (HR, 2.06 (1.314–3.228), *p* = 0.001; HR, 1.694 (1.083–2.648), *p* = 0.023, respectively) (Miari et al., 2021; Xiao et al., 2021). The Kaplan–Meier curves validated the poor prognosis of several immune cells, and the representative cell types are shown in Supplementary Figures S3F–P (log-rank test, cutoff determined by R package survminer). Furthermore, patients of the immune-resistant subtype had a significantly shorter overall survival time than patients of the immune-active subtype (log-rank test, *p* < 0.001, Figure 3H). In general, we discovered that two clinically significant immune subtypes characterized by distinct ir-eRNAs profiles existed in AML, suggesting that ir-eRNAs played vital roles in modulating the immune microenvironment of BM. The immune-resistant subtype was characterized by a high level of suppressive and cancer-promoting immune cells. The immune-active subtype was accompanied by a relatively high level of protective immune cells. Although the absolute abundances of activated T cells, including CD4 T cells and CD8 T cells, were not significant between the two types, fewer suppressive immune cells in the immune-active subtype provided an immune microenvironment with less immune evasion and T-cell dysfunction.

### S100-Enhancer and S100-eRNA May Induce a Poor Immune Microenvironment in AML

**FIGURE 4:**
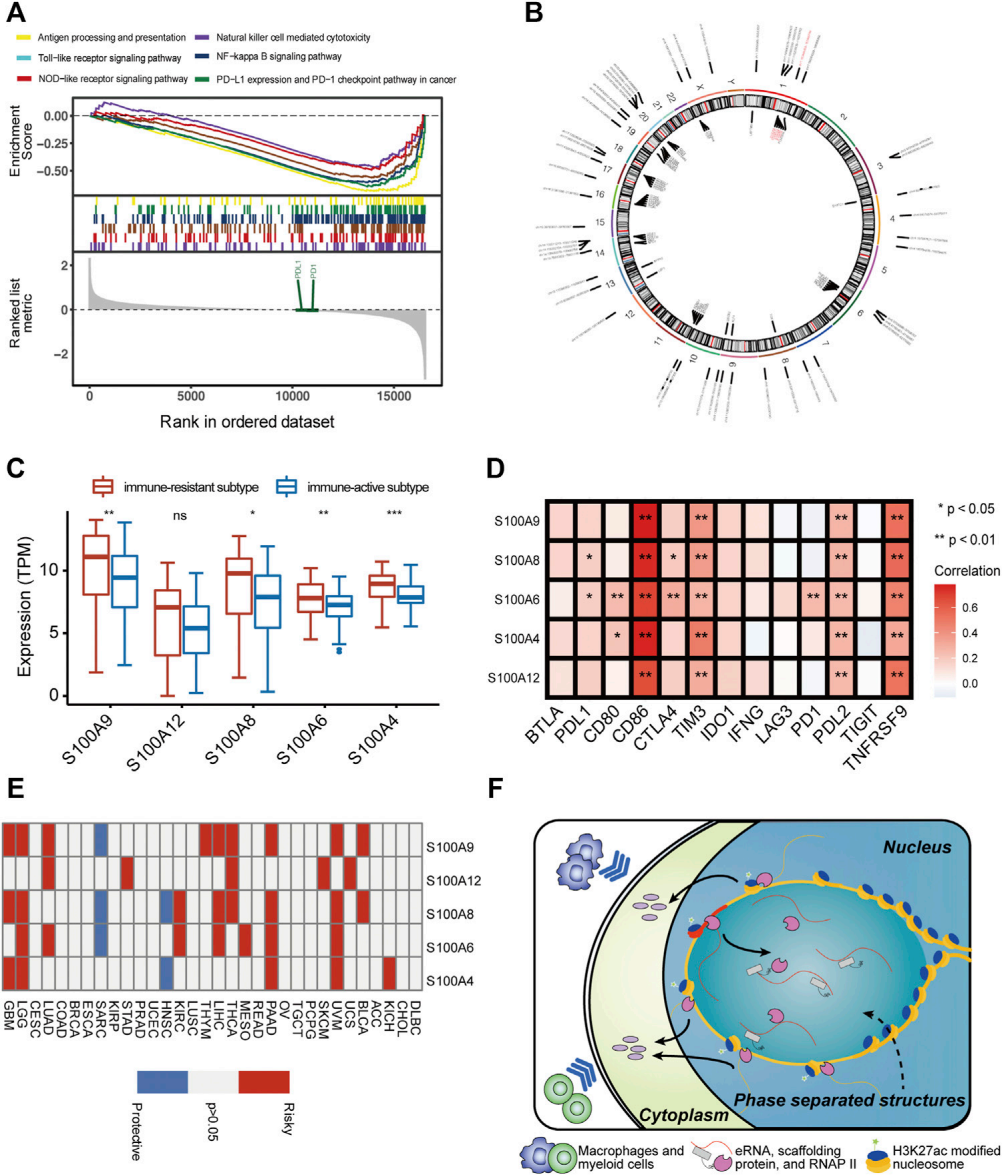
S100-enhancer and S100-eRNA play a role in shaping the immunosuppressive microenvironment. **(A)** GSEA of immune-related pathways confirmed different pathway activation models between immune-resistant and immune-active subtypes. **(B)** The chromosomal locations of potential target genes of ir-eRNAs identified in our study. Red ones were S100-eRNA and five S100 proteins. **(C)** Higher expression level of the nine genes regulated by S100-enhancer and S100-eRNA in the eRNA immune-resistant subtype compared with the immune-active subtype (ns, *p* > 0.05, *p < 0.05; **p < 0.01; ***p < 0.001, Wilcoxon test). **(D)** Correlations between the S100 protein family and immune checkpoint-related genes (Spearman correlation test, *p < 0.05; **p < 0.01). **(E)** The survival relevance of the S100 protein family in pan-cancers (red, risky factors; blue, protective factors; and grey, *p* > 0.05). **(F)** Schematic illustration of the putative mechanism by which S100-enhancer regulates immune infiltration. A phrase-separated structure is shown.

**FIGURE 5:**
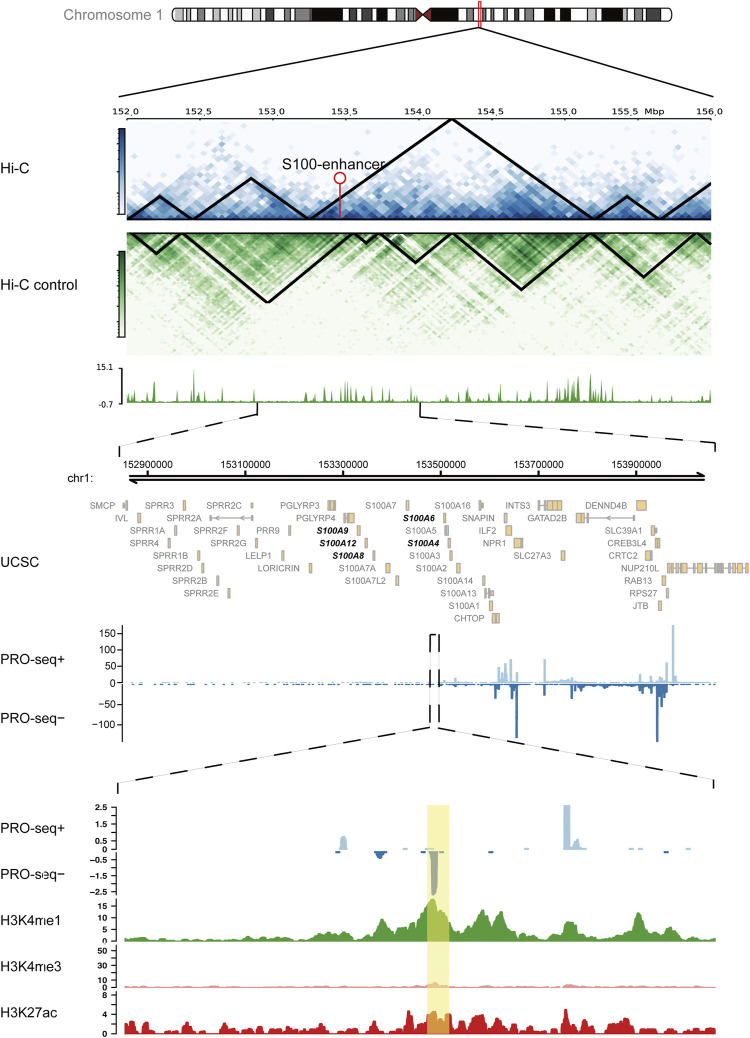
S100-enhancer and correlated genes in the same transcription activation domain (TAD). Upper panel: Hi-C analysis of the genomic region containing the S100-enhancer gene in the K562 cell line, compared with the control cell line. Arrow pointed out the enhancer location. Medium panel: UCSC gene annotations and PRO-seq analysis within ±0.5 Mb region of the S100-enhancer gene, including five correlated genes located in the same TAD (bold). Lower panel: PRO-seq analysis and enhancer-related epigenomic modification analysis (H3K4me1, H3K4me3, and H3K27ac) within the S100-enhancer gene region. S100-enhancer is highlighted in yellow.

Based on the abovementioned findings, we applied the GSVA algorithm to explore pathways that differed between two immune subtypes. The result showed that the immune-resistant subtype was highly associated with innate immune response pathways, while the immune-active subtype was related to the T-cell receptor signaling pathways (Supplementary Figure S4A). The GSEA of immune-related pathways further confirmed that the NF-κB pathway and other innate immune response pathways were highly enriched in immune-resistant subtypes (Figure 4A). Surprisingly, the PDL1 expression and PD1 checkpoint pathway showed a close connection to the immune-resistant subtype, leading to the following analysis to predict the response to ICI therapy. To further explore the possible biological roles of each immune subtype, we identified 1,057 ir-eRNA-related differentially expressed genes (DEGs) between immune-resistant and immune-active subtypes by the limma package (Supplementary Table S3). To investigate whether the DEGs directly correlated with ir-eRNAs, we subsequently screened genes with correlation index >0.3 in the approximately 1 Mb adjacent regions of ir-eRNAs (Zhang et al., 2019; Zhang et al., 2021) and identified 148 potential target genes (Supplementary Table S4). The Venn diagram illustrated that 55 genes showed both different expression levels between two clusters and correlations with ir-eRNAs, which suggested that these genes might be directly activated by immune-related enhancers (Supplementary Figure S4B). Most of these genes were reported as immune-related genes in AML (Li et al., 2020; Brenner and Bruserud, 2018; Borrego, 2013). Among these genes, we focused on five genes (S100A4, S100A6, S100A8, S100A9, and S100A12) from the S100 protein family that was widely reported in AML. The five genes were correlated to a specific eRNA transcribed from a chromosomal region (chr1:153488459–153488748) (supplementary table 4, Figure 4B). We defined this enhancer and eRNA as S100-enhancer and S100-eRNA, respectively. In addition, Supplementary Figure S4C indicates the compact correlation among the five genes. Figure 4C reveals that the five genes exhibited consistent higher expression in the immune-resistant subtype (S100A4 *p* < 0.001, S100A6 *p* = 0.001, S100A8 *p* = 0.014, S100A9 *p* = 0.006, S100A12 *p* = 0.056, Wilcoxon test). Brenner and Bruserud (2018) summarized that the S100 protein family promoted tumor growth by enhancing a suppressive immune microenvironment in AML. Thus, we examined the relationship between S100-eRNA and immune cells and found a strong correlation with suppressive immune cells, including MDSC, macrophage, and regulatory T cell, as was expected (Supplementary Figure S4D). Figure 4D demonstrates that the S100 protein family tightly correlated with the immune checkpoint-related gene expression, suggesting the potential efficacy in predicting response to ICI treatment. Because S100 proteins were positively correlated with the immune-resistant subtype, a relatively poor prognosis would be observed. Supplementary Figures S4E–I demonstrate the hypothesis. We then expanded the S100 protein family to pan-cancer survival analysis. S100 proteins represented general risky factors in the pan-cancer scale, except for S100A6/A8/A9 in Sarcoma (SARC) and S100A4/A8 in Head and Neck Squamous Cell Carcinoma (HNSC) (univariate regression analysis, Figure 4E). These results suggested that the S100-enhancer and S100-eRNA generally facilitated a suppressive inflammatory microenvironment, resulting in a worse outcome via the S100 protein family and related pathway, as Figure 4F illustrates. The illustration of the genomic region revealed that the S100-enhancer and correlated genes were in the same transcription activation domain (TAD) in leukemia cells, which suggested S100-enhancer interacted with nearby genes and induced their transcription (Figure 5). Additionally, PRO-seq demonstrated an active transcription event in the enhancer region, and histone modifications confirmed the epigenomic characteristics of S100-enhancer.

### Immune Infiltration Patterns Indicate a Novel Clinical Classification and Potential Response to Immunotherapy

**FIGURE 6:**
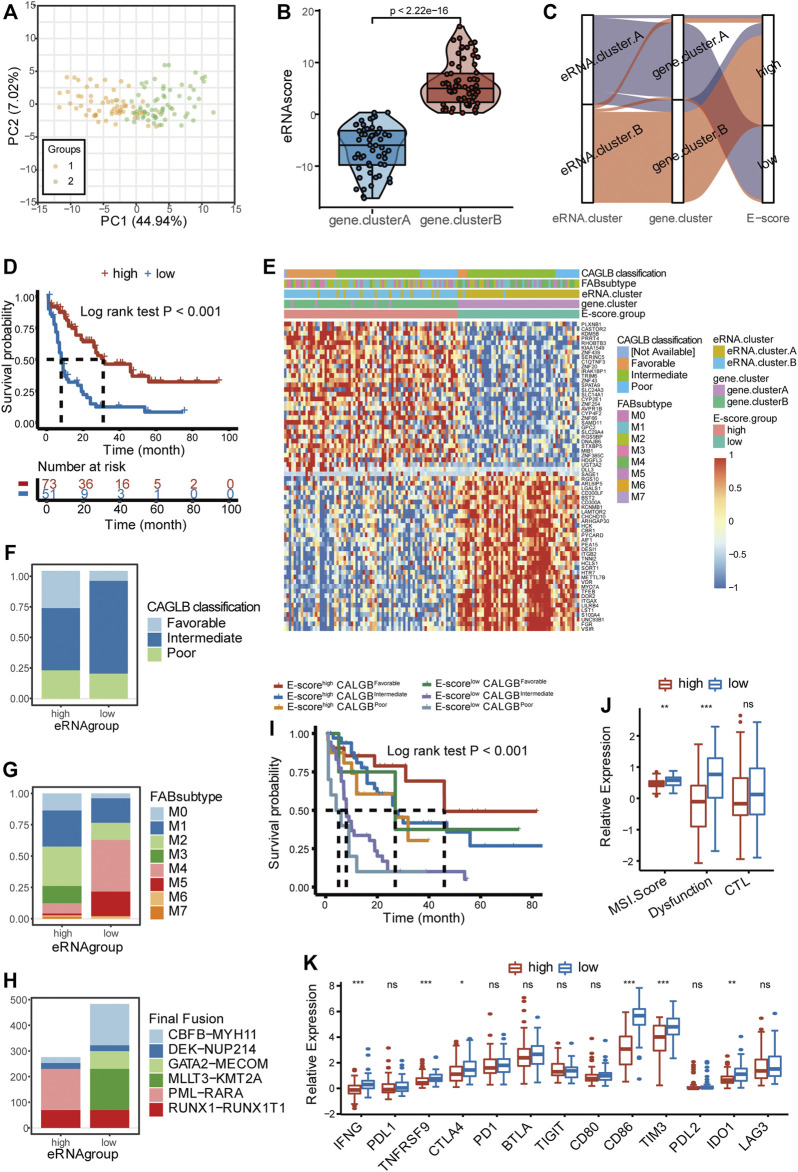
Clinical classification with eRNA and prediction of response to immunotherapy. **(A)** Principal component analysis showed distinguished characteristics between the two gene clusters determined by 66 signature genes. **(B)** Two gene clusters showed distinct eRNA-derived score (E-score) (Wilcoxon test, *p* < 2.22 × 10^−16^). **(C)** Stratifications according to eRNA clustering, gene clustering, and E-score, as well as the correlation among the three stratifications. **(D)** Survival analysis showed different survival outcomes between two E-score groups (log-rank test *p* < 0.001). **(E)** Gene expression heatmap and clinical characteristics (CAGLB risk classification, FAB classifications) in high- and low-E-score groups. **(F)** Distribution of CAGLB risk classification groups (favorable, intermediate, and poor) in high- and low-E-score groups. **(G)** Distribution of FAB subtypes (M0–M7) in high- and low-E-score groups. **(H)** Distribution of fusion types in high- and low-E-score groups. **(I)** Patient stratification based on the CALBG risk classification (favorable, intermediate, and poor) and E-score group (high and low) and corresponding prognosis (log-rank test *p* < 0.001). **(J)** Enrichment of immunotherapy-related markers in high- and low-E-score groups (ns, *p* > 0.05, *p < 0.05; **p < 0.01; ***p < 0.001, Wilcoxon test). **(K)** Expression of immune checkpoints and related genes in two E-score groups (ns, *p* > 0.05, *p < 0.05; **p < 0.01; ***p < 0.001, Wilcoxon test). eRNA.cluster.A and gene. cluster.A, immune-resistant subtype; eRNA.cluster.B and gene. cluster.B, immune-active subtype.

To explore the roles of immune microenvironment patterns in predicting clinical prognosis and potential response to immunotherapy, we created a gene signature based on prognostic DEGs between immune-resistant and immune-active subtypes (246/1,057 prognostic DEGs, Supplementary Table S5). Random Forest regression, a machine learning-based algorithm, was used to get representative genes (66/246 after Random Forest regression, Supplementary Table S5). We then performed an unsupervised clustering analysis with the 66 signature genes and obtained two gene clusters (Supplementary Figures 5A–D). The immune infiltration and survival analysis based on both ir-eRNAs and signature genes were similar, thus supporting the robustness of immune subtypes (Supplementary Figures 5E–G). The result of PCA revealed the well-defined characteristics of immune subtypes as well (66 signature genes, Figure 6A).

The abovementioned results demonstrated that ir-eRNA alteration had a significant impact in generating distinct TME patterns. These findings, however, were limited to the population level and could not properly predict the immune profiles of AML at the individual level. Given the alternative heterogeneity and diversity of ir-eRNA-mediated immune profiles, we developed a scoring system based on these phenotype-related genes to quantify the TME pattern of individual patients in AML through the PCA algorithm, which was named eRNA-derived score (E-score) (see Methods, Figure 6B). E-score well distinguished the two immune subtypes as was expected (*p* < 0.001, Wilcoxon test; Figures 6B,C). The high-E-score group had a better prognosis than the low-E-score group (log-rank test, *p* < 0.001, Figure 6D). Nearly all immune-active subtypes were stratified into the high-E-score group (Figure 6C).

AML was commonly classified by the French–American–British (FAB) classification and the morphology, immunology, cytogenetics, and molecular biology (MICM) classification via genetic, immunophenotypic, pathological, and clinical features. CALGB cytogenetics-based risk classification and ELN 2017 risk classification provided widely used predictive models based on the integration of cytogenetics and mutational status (Döhner et al., 2017; Tallman et al., 2019). We examined the efficacy of E-score in distinguishing various clinical characteristics by the TCGA cohort and another independent cohort, Beat AML. It was found that the high- and low-E-score groups showed distinct clinical characteristics (Figure 6E). When favorable CALGB risk patients were almost concentrated in the high-E-score group, the low-E-score group contained a greater proportion of intermediate and poor CALGB risk patients (Figure 6F). The M3 subtype was categorized exclusively into the high-E-score group, while M4 and M5 subtypes were most concentrated in the low-E-score group (Figure 6G). Furthermore, PML-RARα translocations only existed in the high-E-score group (Figure 6H). On the contrary, CBFB-MYH11, GATA2-MECOM, and MLLT3-KMT2A only occurred in the low-E-score group (Figure 6H). Differences in other clinical features, including the response to chemotherapy, ELN2017 risk classification, and WHO classification, are shown in Supplementary Figures 5H–J. The group with both low E-score and intermediate/poor risk showed poor prognosis compared with the other groups, which indicated that a good proportion of patients with intermediate/poor risk showed relatively better outcomes (Figure 6I). We have mentioned that the immune-resistant subtype was enriched in the PD−L1 expression and PD−1 checkpoint pathway (Figure 4A). The immune-resistant TME with abundant suppressive immune microenvironment generally adopted a better response to immune checkpoint therapy, such as the anti-PD1/PDL1 agents, in solid tumors (Turan et al., 2018). We predicted the response to immunotherapy in the two groups. The Microsatellite Instability (MSI) score and T-cell dysfunction score of Tumor Immune Dysfunction and Exclusion (TIDE) were higher in the low-E-score group, suggesting a predictive better response to immunotherapy (Figure 6J). We also detected other non-redundant immune checkpoints and checkpoint-related gene expression between the two clusters (Figure 6K). Several checkpoint molecules, including IFNG, TNFRSF9, CTLA4, CD86, TIM-3, and PD-L2, had significantly increased expression in the low-E-score group, and their effects on T-cell dysfunction and immune escape have been verified in AML (Hobo et al., 2018; Kikushige, 2021). However, there was no significant difference, but only a trend, in the expression of PD1 and PDL1, implying that the TME modulation is complicated and understudied. On the other side, our result partly explained the reason why current clinical trials of anti-PD1 agents exhibited controversial results. A group of intermediate/poor-risk patients with low E-score might be the potential population benefiting from the ICI treatment, which requires clinical trials for further validation. Based on the aforementioned results, our E-score system firstly proposed a quantizable system to predict response to ICI therapy in AML.

### E-Score Associated With Genetic Risk Stratification Serves as an Independent Prognostic Factor in AML

**FIGURE 7:**
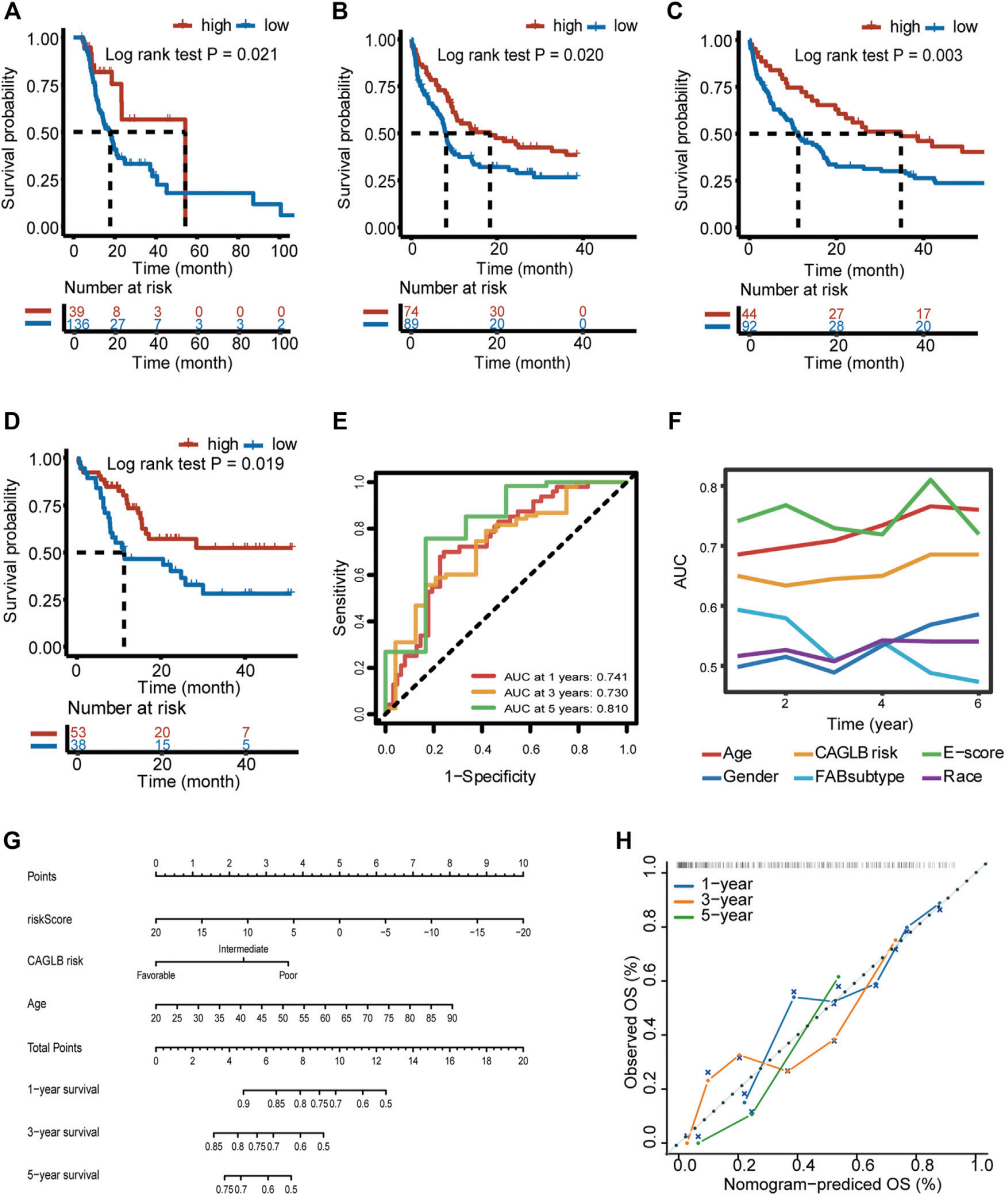
Prognostic power of E-score and a prognostic nomogram established. **(A–D)** Survival difference between high- and low-E-score groups validated in the Beat AML **(A)** (log-rank test *p* = 0.021), GSE37642 **(B)** (log-rank test *p* = 0.020), GSE12417 **(C)** (log-rank test *p* = 0.003), and GSE10358 **(D)** (log-rank test *p* = 0.019) cohort. **(E)** Predictive power of E-score for 1-, 3-, and 5-year survival, evaluated by AUC (0.741 at 1 year, 0.730 at 3 years, and 0.810 at 5 years). **(F)** Predictive power of E-score for overall survival compared with other clinical characteristics, evaluated by AUC. **(G)** The nomogram constructed by integrating E-score and other clinical features. **(H)** Calibration curves of our nomogram on the estimation of 1-, 3-, and 5-year overall survival.

To discover whether E-score could provide satisfactory predictive efficacy in AML, we proved that E-score was an independent prognostic factor in 4 validation cohorts (Beat AML, GSE37642, GSE12417, and GSE10358). Consistent survival differences were observed in all the validation cohorts mentioned above (Beat AML cohort, log-rank test, *p* = 0.021; GSE37642, log-rank test, *p* = 0.020; GSE12417, log-rank test, *p* = 0.003; and GSE10358, log-rank test, *p* = 0.019; Figures 7A–D). Both univariate cox regression and multivariate cox regression confirmed the independent predictive power of age, CAGLB risk classification, and E-score (TCGA-LAML cohort, Supplementary Figure 6A). Log-rank regression of these clinical features also revealed that age and CAGLB risk classification were independent predictors (TCGA-LAML cohort, Supplementary Figures 6B–F). E-score showed a high area under the curve (AUC) in a time sequence (AUC_1year_ = 0.741, AUC_3year_ = 0.730, AUC_5year_ = 0.810, Figure 7E). Additionally, compared with other clinical characteristics (gender, age, race, FAB subtype, and CAGLB risk classification), the E-score exhibited the best capability at predicting OS in the TCGA cohort (Figure 7F). Given that the E-score showed an excellent predict power in OS, we constructed a nomogram integrating E-score with other clinical features (TCGA-LAML cohort as the training cohort, Figure 7G). The calibration curves of the nomogram for 1-year, 3-year, and 5-year survival probability exhibited excellent consistency with the ideal performance, indicating the high accuracy of our nomogram (TCGA-LAML cohort, Figure 7H). The nomogram discrimination as evaluated by C index was 0.714 (95% CI = 0.651–0.777, *p* < 0.0001), which was superior to that of the E-score alone (0.674, 95% CI = 0.608–0.740, *p* < 0.0001), CAGLB risk classification (0.594, 95% CI = 0.526–0.663, *p* < 0.0001), and age (0.661, 95% CI = 0.596–0.726, *p* < 0.0001) in the TCGA-LAML cohort. The nomogram was externally confirmed in the validation cohort, the Beat AML cohort. The C index was 0.684 (95% CI = 0.614–0.755, *p* < 0.0001; Beat AML cohort), indicating a significant discriminative ability. The calibration curve presented a good agreement for 1-year OS rates in the validation cohort (Supplementary Figures 6G). However, calibration curves exhibited underestimated 3- and 5-year OS rates (Supplementary Figures 6H–I). In all, the E-score system had outstanding predict power in both OS and response to ICI therapy, which improved the current predictive models and made it potential for clinical practice.

## Conclusion

This study identified immune-related prognostic eRNAs (ir-eRNAs) for AML. Clustering of ir-eRNAs revealed two distinct immune subtypes, the immune-resistant subtype and the immune-active subtype. S100-eRNA was found to lead to worse outcomes with the activation of the S100 protein family and subsequently induced a suppressive TME. A novel eRNA-derived scoring system (E-score) was developed and showed great stratification power for patients in terms of the immune microenvironment and clinical characteristics. Finally, we established a prognostic nomogram with satisfactory accuracy by integrating E-score and other clinical features. This study provided a comprehensive understanding of the impact of eRNAs on shaping the immune microenvironment in AML and developed eRNA-derived tools to predict prognosis.

## Discussion

In this study, we identified that AML bone marrow samples can be classified into immune-resistant and immune-active subtypes by ir-eRNAs. Then, we revealed the underlying rationality of the immune classification by an enhancer–gene pair. Finally, we developed a prognostic model based on immune scoring, E-score, for predicting overall survival and response to immunotherapy in AML patients.

Our study revealed that the high suppressive immune microenvironment with high counts of regulatory T cells and MDSCs correlated with poor prognosis. In this paper, we used eRNA, a non-coding RNA, for effective clustering and suggested that enhancer plays an important role in the construction of the BM microenvironment. We cautiously inferred the conclusion based on the fact that eRNAs are excellent markers for active enhancers that facilitated the transcription of target genes (Andersson et al., 2014). We demonstrated that a large amount of eRNAs was correlated with the abundance of immune cells in AML. Moreover, the putative genes in the vicinity of these ir-eRNAs have been experimentally validated to correlate with the invasion and progression of AML (Borrego, 2013; Brenner and Bruserud, 2018; Li et al., 2020). Bulk-level RNA-seq data of the current public database were the confounding profiles of the different cell types, which compromised the power of mRNAs to predict the molecular and clinicopathological features of tumor cells. The essential TME-promoting mRNAs expressed in AML blasts could be averaged by the background expression of other cell types in the TME. However, eRNA has been reported to associate with tumor traits, such as response to immunotherapy, more powerfully because of the strong cell lineage specificity (Chen and Liang, 2020). E-score derived from eRNA classification had a higher AUC (0.810 vs. 0.68) compared to other risk scores derived from immune cell abundance as a predictor of OS (Wang et al., 2021), implying that eRNA-based immune subtypes had better efficacy to predict the OS of AML patients.

The interaction between tumor cells and the microenvironment together promoted tumorigenesis and tumor progression. The S100 protein family provided a suppressive immune microenvironment in a variety of tumors, promoting tumorigenesis and metastasis (Brenner and Bruserud, 2018). Among the genes with a high correlation with S100-eRNA, our attention was focused on S100A8/A9, a heterodimer that is overexpressed in many cancers and associated with suppressive TME. S100A8/A9 is an activator of monocytes and macrophages and leads to neutrophil infiltration, correlating with a poor prognosis of patients. S100A8/A9 promotes tumor development and invasiveness by increasing leukemic cell growth through RAGE and secretion of pro-inflammatory cytokines through the TLR4-NFκB pathway (Hiratsuka et al., 2008). Kaikkonen et al. (2013) have reported that eRNA takes part in the activation of the TLR4-NFkB pathway in macrophages by promoting enhancer–promoter looping and regulating mediator recruitment, which increases our confidence in that the S100-eRNA-S100 protein family axis is potentially involved in shaping the immune microenvironment.

In this study, we first systematically concluded the prediction of response to immunotherapy linked to immunophenotyping in AML. Several clinical trials about checkpoint inhibitors therapy have been pursued in AML and got controversial conclusions (Ghosh et al., 2020). Identification of specific populations who might benefit from ICI therapy would be the ideal strategy to solve this problem. Our study firstly developed a method to predict the potential population that benefited from ICI therapy and implied that other ICIs, including TIM-3, might be more attractive targets. We collected general predictors of response to ICI therapy. T-cell exclusion score was excluded due to suspiciously overestimated MDSCs. In addition, the tumor mutation burden (TMB) was also omitted because TMB is generally quite low in AML. Further validation of our model requires further detailed survival data of the immunotherapy cohort. Our results suggest E-score may be a promising predictor for the choice of immunotherapy, especially for M4/M5 patients with high E-score and abundant suppressive immune infiltration.

It is important to note that limitations existed in our study. First, because of the similarities between leukemia cells and myeloid-derived cells such as MDSC and monocytes, a possible overestimation of MDSC and monocyte existed. We carefully based the conclusion on the abundance of these cells. Whether eRNA functions as an initiation factor of vital gene transcription needs further functional experiment because eRNA may be only a marker of enhancer active transcription instead of a functional factor. The enhancer–gene pairs were determined by co-expression analysis as in other studies (Zhang et al., 2019), which was the best strategy for large clinical cohorts currently. More advantages of single-cell RNA-seq or single-cell ATAC-seq could improve the accuracy of prediction and support further analysis.

In conclusion, our study distinguished AML into immune-resistant and immune-active subtypes by ir-eRNA for the first time. The important roles of these eRNAs in the clinic require investigation, which may provide new insight into the pathogenesis of AML. The predictive model with high accuracy provides new tools for physicians. This study increases the understanding of the important roles of enhancers on the BM microenvironment and might provide novel therapeutic targets.

## Data Availability

The original contributions presented in the study are included in the article/Supplementary Material, further inquiries can be directed to the corresponding authors.

## Abbreviations

eRNA, enhancer RNA; TME, tumor microenvironment; TCGA, The Cancer Genome Atlas; GEO, Gene Expression Omnibus; DEG, differentially expressed gene; FDR, false discovery rate; KEGG, Kyoto Encyclopedia of Genes and Genomes; AML, acute myeloid leukemia; MDSC, myeloid-derived suppressive cell; ir-eRNA, immune-related eRNA; HRs, hazard ratios; GSVA, gene set variation analysis; ssGSEA, single-sample gene set enrichment analysis; and PCA, principal component analysis.
