# Supplementary figures and images for: eRNAs Identify Immune Microenvironment Patterns and Provide a Novel Prognostic Tool in Acute Myeloid Leukemia
Source: Front Mol Biosci. 2022 May 2;9:877117. doi: 10.3389/fmolb.2022.877117 (PMC9108177; doi:10.3389/fmolb.2022.877117)

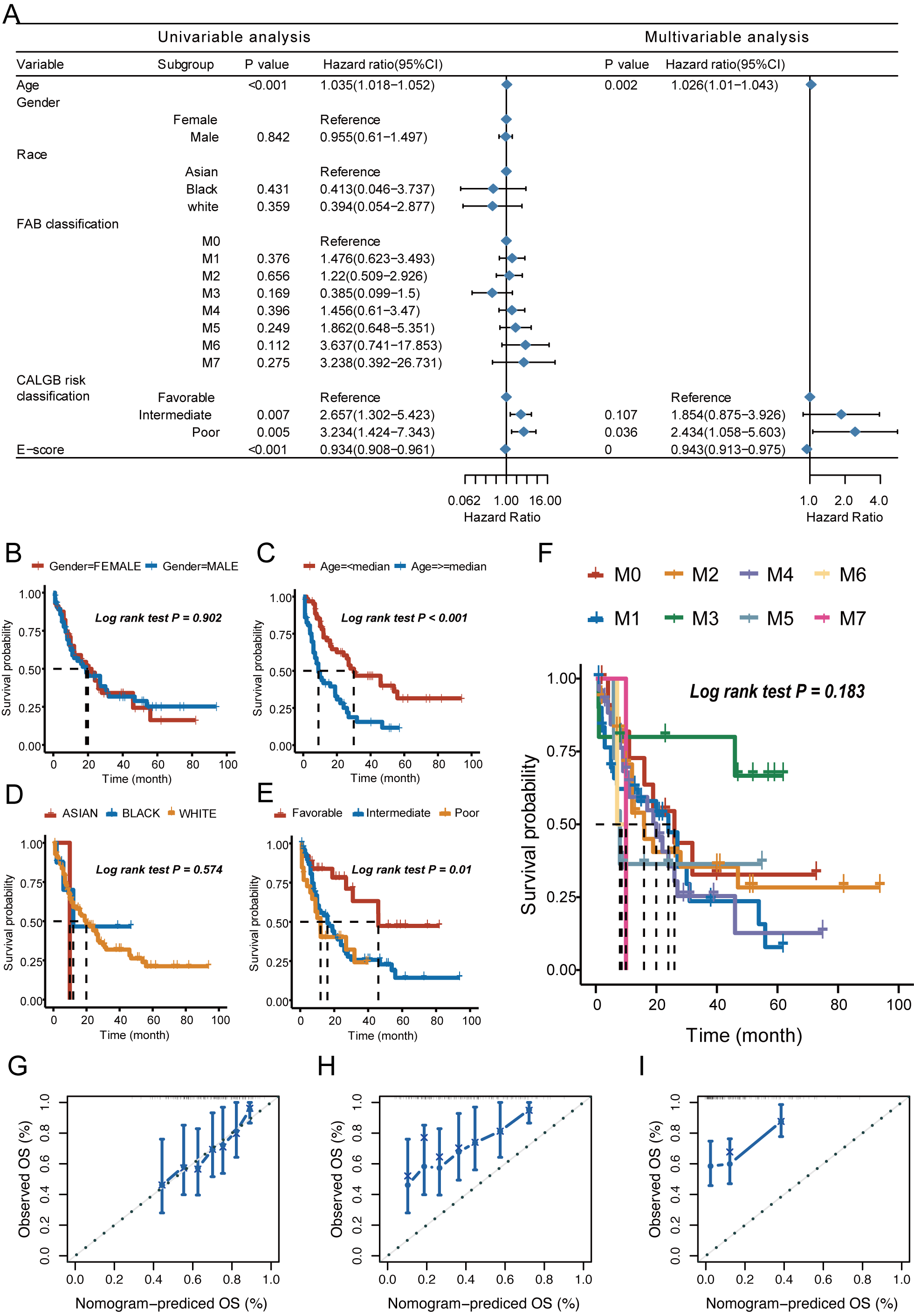

Supplement: Supplementary file 1 [file Image6.TIF]

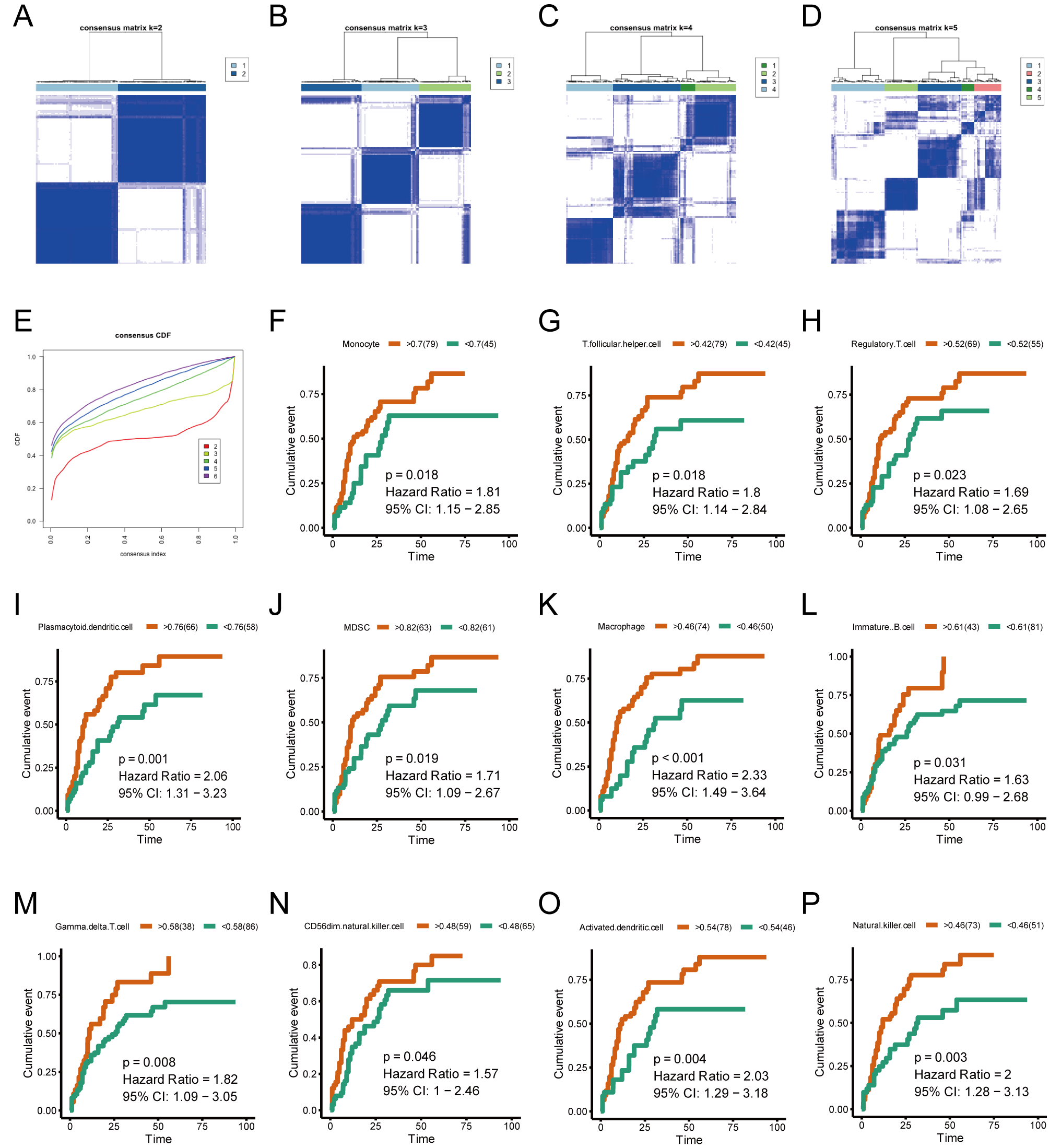

Supplement: Supplementary file 2 [file Image3.TIF]

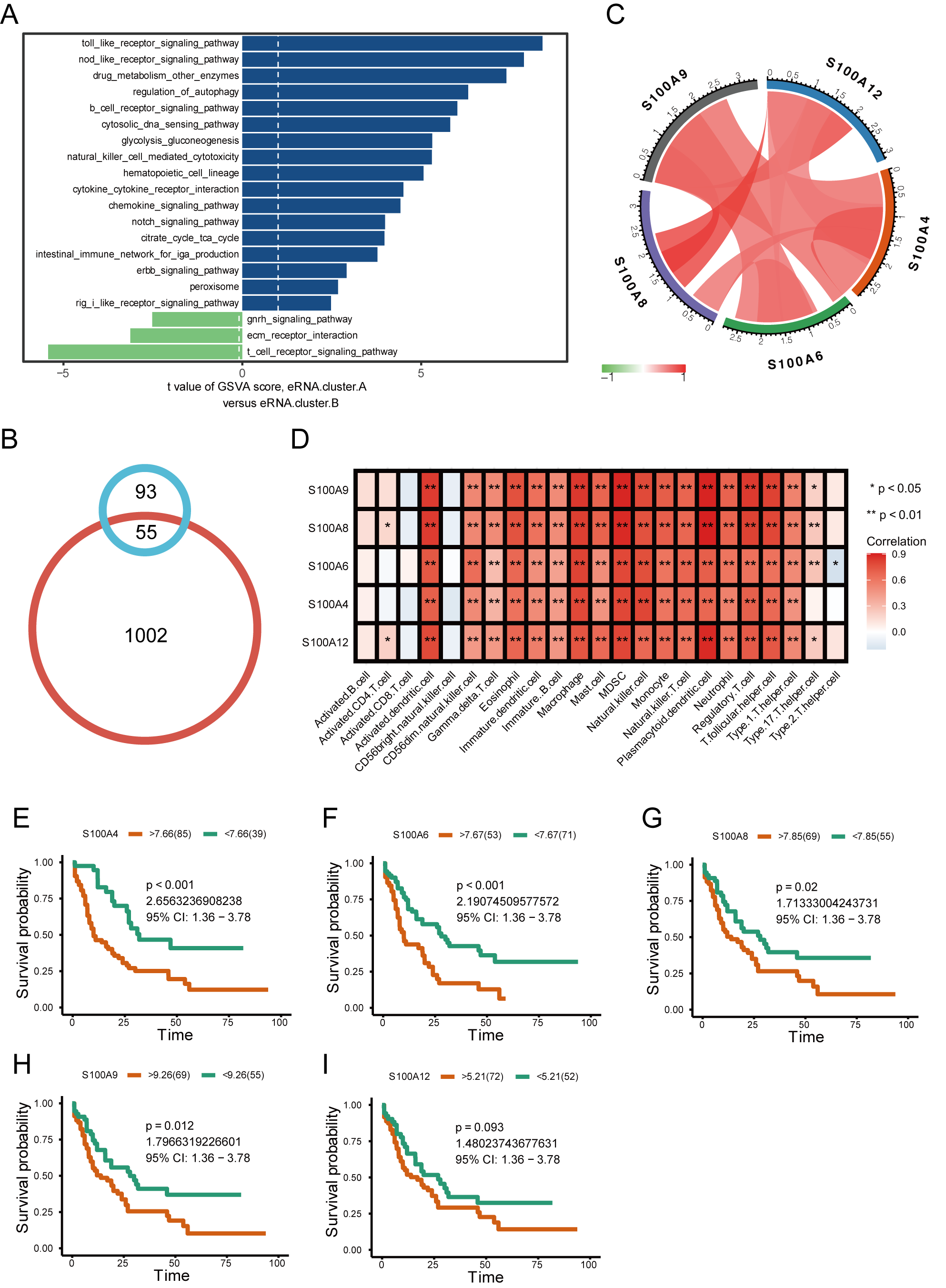

Supplement: Supplementary file 3 [file Image4.TIF]

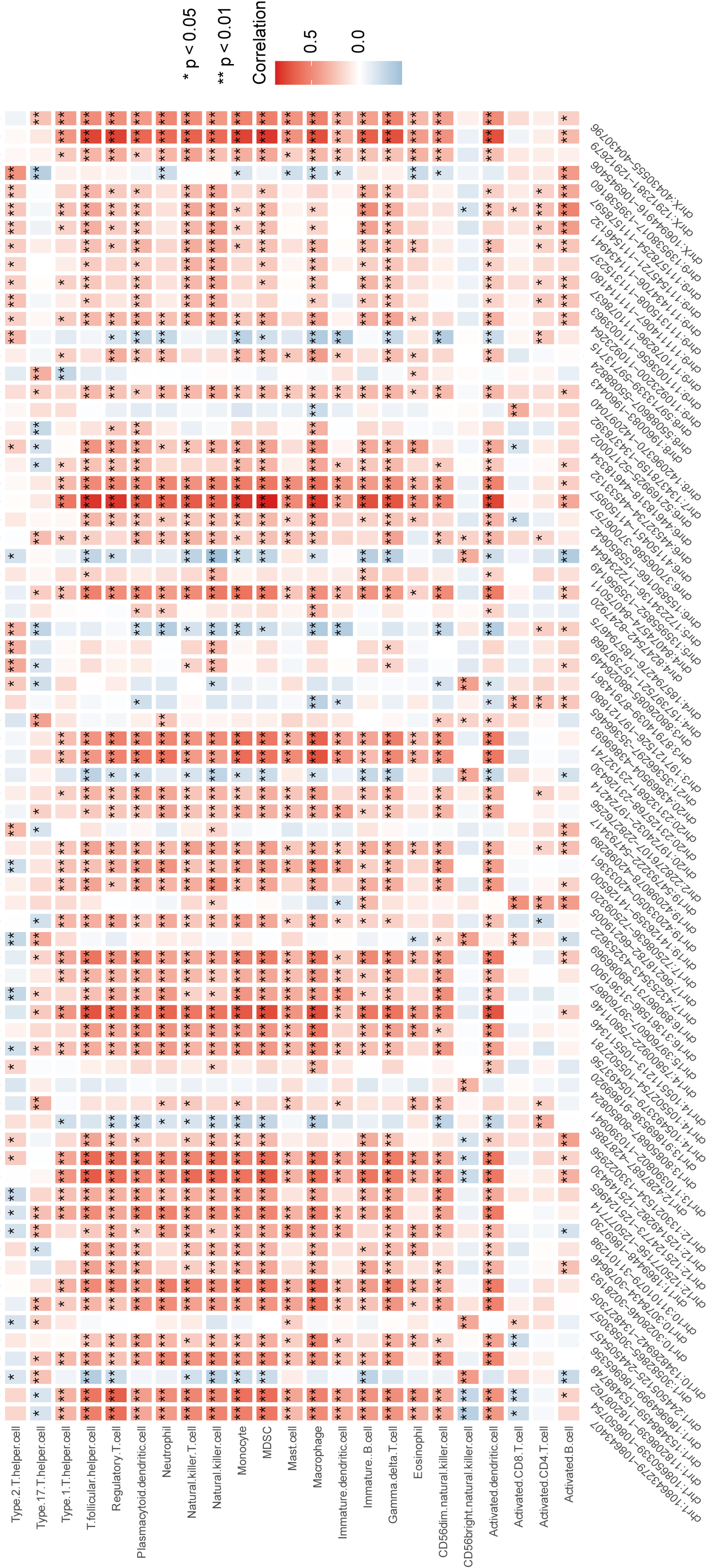

Supplement: Supplementary file 4 [file Image2.TIF]

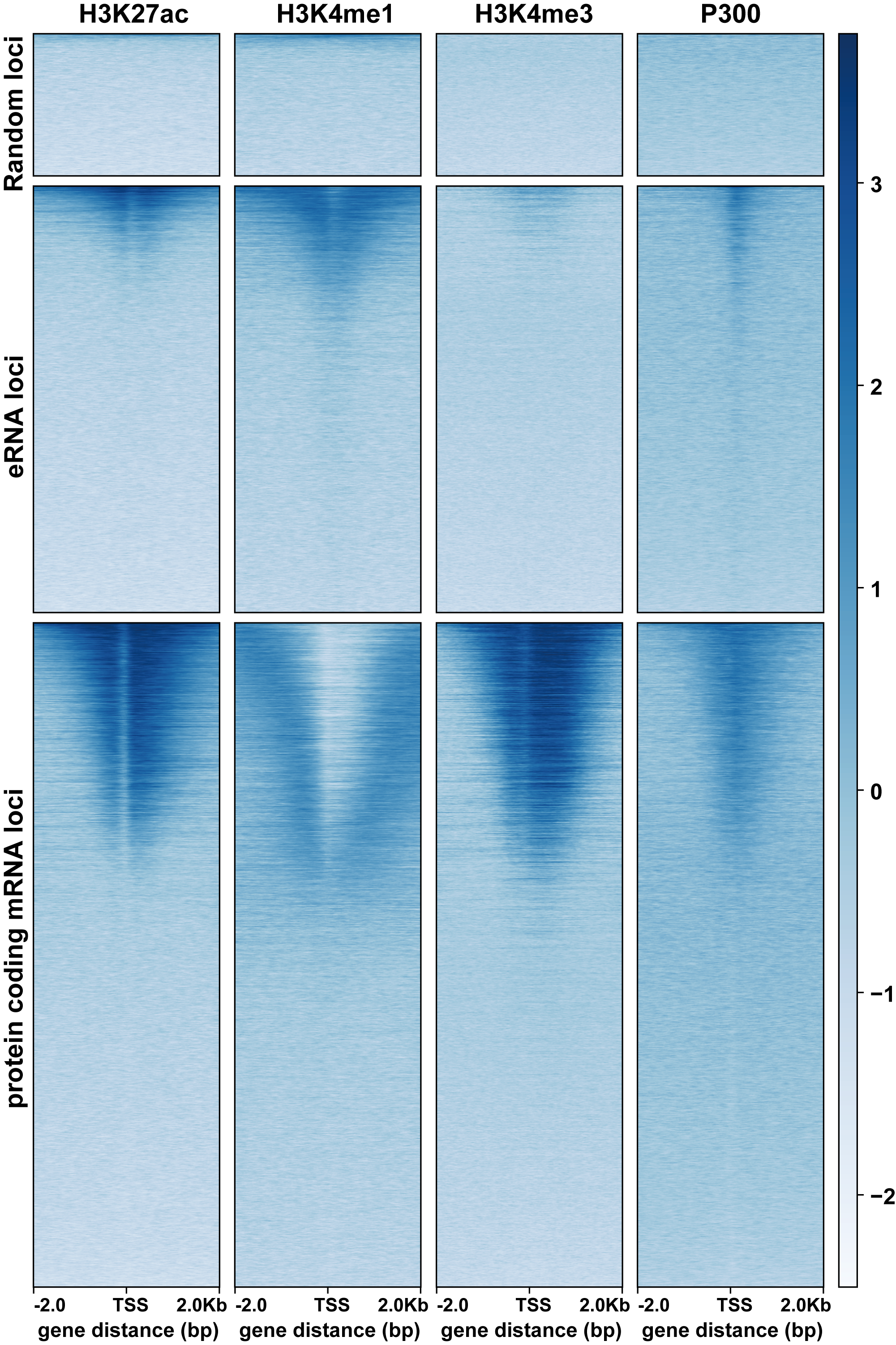

Supplement: Supplementary file 5 [file Image1.TIF]

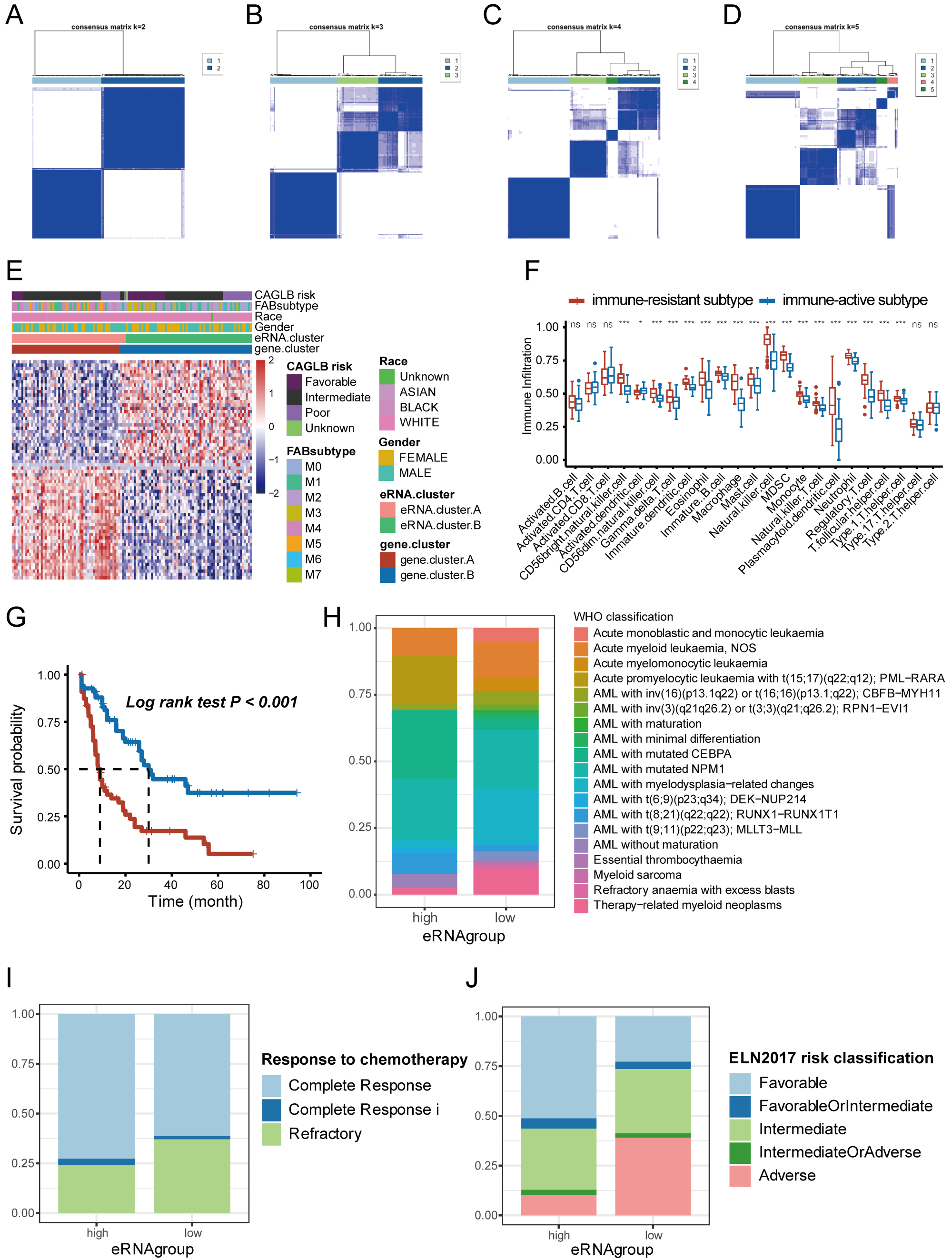

Supplement: Supplementary file 7 [file Image5.TIF]
